# ! !*Words fail to supply a title. Reference is made herein to the report of the proceedings of the American Institute of Homœopathy as given in the *Medical Advance* of July, 1887.

**Published:** 1887-10

**Authors:** H. Hitchcock

**Affiliations:** Newark, N. J.


Lippe! Wells! Bayard! Ye members of the I. H. A.! Avaunt! Go hide your heads for shame! Go bury yourselves in the deepest depths of the underworld! Can ye read the "masterly effort which held the vast audience throughout," and the words of wisdom, science, and truth which follow, and dare to show your heads again? Shame on ye, "who think we should follow the teachings of a 'master' of half a century ago," and do not "keep abreast of the tenets and teachings of more modern times, using the measures of any and every school when available." Ye are old enough to know better! Ye poor fools! *Ye* claim to teach and practice Homœopathy? How absurd! It has been said that he who would teach must first learn himself. Ye are half a century behind the times! Go learn of your MASTERS of to-day! You will find them in the INSTITUTE! Shame on ye, ye old fossils! Ye have shown no signs of "PROGRESS," and your years are too many to begin now! Ye still adhere to a "FALLACY"! Ye still adhere to "EXCLUSIVISM"! "SECTARIANISM" is still one of your greatest follies!

---

* Words fail to supply a title. Reference is made herein to the report of the proceedings of the American Institute of Homœopathy as given in the *Medical Advance* of July, 1887.

If ye cannot come out from your shells and shout and yell and cry and whine and plead for RECOGNITION, the sooner we are rid of you and your clan the better! Mark ye this: "The American Institute of Homœopathy adheres, as it has always done, to its *object*, as declared by its founders in the first article of its Constitution, namely: 'The improvement of homœopathic therapeutics, and *all other* departments of medical science,' and it is proud of its achievements up to this time."

Whoop la! Look at that for a "masterly effort"! Didn't you have something to do with the founding of the Institute? What did you silly creatures run away for, and start up a "mutual admiration society" of your own? Assuredly, THIS is proof enough that you are not "abreast of the tenets and teachings of modern times."

"'Tis true, 'tis pity; and pity 'tis, 'tis true."

Why don't you yell for HARMONY and RECOGNITION? Subscribe at once for the *Cyclopædia of Drug Pathogenesy!* Throw your Hahnemann, Jahr, Bœnninghausen, and Hering in the fire! Get Dake, Dudgeon, Burt, Hughes & Co. instead; be sensible and rational.

Use ten drops of tincture of Aconite, with a four-ounce solution of Gum Arabic for asthma! Throw aside your silly prejudices against pessaries—"personal experience will show their importance." Remember, that topical applications and "local action" of drugs are absolutely essential.

Suppression of eczema is a fad! External applications of astringents and other drugs do not produce subsequent ill results. Thus saith the sapient! The idea! "How can doses of Sulphur affect a parasite that grows underneath the skin, and that can only be killed by some topical application?" It's absurd! "Catch your rabbit before you skin him."

Last of all, know this: "CORRECT DIAGNOSIS" is "THE FIRST ESSENTIAL." Your ideas about symptomatology are simply—bosh!

H. HITCHCOCK.

NEWARK, N. J., July 19th, 1887.

